# Physical and Cognitive Correlates of GPS-Derived Life-Space Characteristics in Older Adults

**DOI:** 10.1093/geroni/igab046.095

**Published:** 2021-12-17

**Authors:** Kyle Moored, Breanna Crane, Michelle Carlson, Andrea Rosso

**Affiliations:** 1 University of Pittsburgh, Pittsburgh, Pennsylvania, United States; 2 Johns Hopkins Bloomberg School of Public Health, Baltimore, Maryland, United States; 3 Johns Hopkins University, Baltimore, Maryland, United States

## Abstract

Life-space mobility, movement within one’s living environment, is important for functional independence in later life. It is unclear which life-space characteristics (i.e., space, duration, shape) are most affected by physical and cognitive limitations. GPS-derived measures mitigate recall bias and offer novel ways to characterize life-space. We examined associations between physical and cognitive performance and GPS-derived life-space characteristics. Participants were 164 community-dwelling adults (Age: M=77.3±6.5) from baseline data of a clinical trial to improve walking in older adults. Participants carried a portable GPS for 7 days, which passively collected real-time location. Standard deviational ellipses (SDEs) and minimum convex polygons (MCPs) were derived for each day. Area and compactness of these measures quantified activity space and shape, respectively. For each measure, 7-day medians and median absolute deviations (MAD) were computed to capture both central tendency and variability of weekly activity. Activity duration was quantified as percentage of time outside home. Adjusting for age and sex, percent time outside home was associated with lower mobility performance (i.e., 6-minute walk (6MWT), figure 8 walk, ρ’s=.17-.18, p’s<.05) and executive functioning (i.e., Trail Making Test, Part A: ρ=.16, p=.04, Part B: ρ=.19, p=.01). Median MCP and SDE areas, but not compactness, were associated with 6MWT performance (ρ’s=.18-.20, p’s<.05). MCP area MAD was associated with greater global cognition (3MSE, ρ=.15, p=.05). Life-space characteristics were differentially associated with performance measures, suggesting physical and cognitive limitations may constrain life-space mobility via different mechanisms. Variation in these associations by neighborhood walkability and active versus passive travel will also be examined.

